# CFIR simplified: Pragmatic application of and adaptations to the Consolidated Framework for Implementation Research (CFIR) for evaluation of a patient‐centered care transformation within a learning health system

**DOI:** 10.1002/lrh2.10201

**Published:** 2019-09-26

**Authors:** Nadia Safaeinili, Cati Brown‐Johnson, Jonathan G. Shaw, Megan Mahoney, Marcy Winget

**Affiliations:** ^1^ Division of Primary Care and Population Health Stanford University School of Medicine Palo Alto California

**Keywords:** CFIR, evaluation, learning health system, primary care, patient‐centered care, transformation

## Abstract

**Introduction:**

The Consolidated Framework for Implementation Research (CFIR) is a commonly used implementation science framework to facilitate design, evaluation, and implementation of evidence‐based interventions. Its comprehensiveness is an asset for considering facilitators and barriers to implementation and also makes the framework cumbersome to use. We describe adaptations we made to CFIR to simplify its pragmatic application, for use in a learning health system context, in the evaluation of a complex patient‐centered care transformation.

**Methods:**

We conducted a qualitative study and structured our evaluation questions, data collection methods, analysis, and reporting around CFIR. We collected qualitative data via semi‐structured interviews and observations with key stakeholders throughout. We identified and documented adaptations to CFIR throughout the evaluation process.

**Results:**

We analyzed semi‐structured interviews with key stakeholders (n = 23) from clinical observations (n = 5). We made three key adaptations to CFIR: (a) promoted “patient needs and resources,” a subconstruct of the outer setting, to its own domain within CFIR during data analysis; (b) divided the “inner setting” domain into three layers that account for the hierarchy of health care systems (i. pilot clinic, ii. peer clinics, and iii. overarching health care system); and (c) tailored several construct definitions to fit a patient‐centered, primary care setting. Analysis yielded qualitative findings concentrated in the CFIR domains “intervention characteristics” and “outer setting,” with a robust number of findings in the new domain “patient needs and resources.”

**Conclusions:**

To make CFIR more accessible and relevant for wider use in the context of patient‐centered care transformations within a learning health system, a few adaptations are key. Specifically, we found success by teasing apart interactions across the inner layers of a health system, tailoring construct definitions, and placing additional focus on patient needs.

## 1 INTRODUCTION

### 1.1 The Consolidated Framework for Implementation Research

The Consolidated Framework for Implementation Research (CFIR) is a comprehensive implementation science framework compiled from 20 sources spanning 13 scientific disciplines.^1^, ^2^ It was developed to guide effective implementation of evidence‐based practices from design to evaluation and is most commonly cited in evaluations of single interventions as opposed to complex transformation initiatives. The CFIR comprised 39 constructs divided into the following five domains:

1. *Intervention characteristics*: aspects of an intervention that may impact implementation success, including its perceived internal or external origin, evidence quality and strength, relative advantage, adaptability, trialability, complexity, design quality and presentation, and cost.
2. *Outer setting*: external influences on intervention implementation including patient needs and resources, cosmopolitanism or the level at which the implementing organization is networked with other organizations, peer pressure, and external policies and incentives.
3. *Inner setting*: characteristics of the implementing organization such as team culture, compatibility and relative priority of the intervention, structures for goal‐setting and feedback, leadership engagement, and the implementation climate.
4. *Characteristics of individuals*: individuals' beliefs, knowledge, self‐efficacy, and personal attributes that may affect implementation.
5. *Process of implementation*: stages of implementation such as planning, executing, reflecting and evaluating, and the presence of key intervention stakeholders and influencers including opinion leaders, stakeholder engagement, and project champions.

The comprehensive and multifaceted nature of CFIR makes it well matched to capture the complexities of transformative interventions such as care model redesigns. Some implementers and evaluators have reported struggling to translate the complex and sometimes repetitive construct definitions to fit their initiatives^1^, ^3^, ^4^; additionally, CFIR's combined breadth and depth is not always feasible for implementation in rapid time frames. Reported use of CFIR in the context of evaluation of multifaceted, patient‐centered care transformations is rare; we know of only a handful of instances, recently reported.^2^, ^4^, ^5^, ^6^

Here, we report adaptations we made to the CFIR in the evaluation of a multicomponent, patient‐centered care transformation, designed to address the Quadruple Aim.^7^, ^8^ The Quadruple Aim of health care expands the Triple Aim's goals^7^ of quality of care, patient experience, and cost savings by adding a provider/staff satisfaction component. We believe our adaptations to CFIR are generalizable to any patient‐facing intervention implemented in an outpatient health care system. We describe our application and adaptation of the framework at each stage of our evaluation**,** along with the resulting outcomes derived from our approach.

## 2 QUESTIONS OF INTEREST

- Is the Consolidated Framework for Implementation Research (CFIR) a useful, accessible tool to use when evaluating a complex intervention within a learning health system?
- Which adaptations are needed to make CFIR better suited to evaluating complex care transformations within a learning health system context?
- How might our experience of and lessons learned from using CFIR to evaluate a complex primary care transformation within a learning health system inform future evaluations of similar interventions?

## 3 METHODS

### 3.1 Setting

Primary Care 2.0 is a patient‐centered care model developed by Stanford Primary Care leaders, with input from numerous stakeholders, including patients and families.^8^, ^9^ Table 1 lists the six modules and their key components which enable focus on health (rather than disease), flexibility in types of appointments, and provision of services beyond traditional primary care.

**Table 1:** Initial Primary Care 2.0 modules and definitions

Team‐based care	Multidisciplinary team led by MDs and nurse practitioner/physician assistant “Advance Practice Providers” (APP)
“Care coordinator” role	Expanded medical assistant role including in‐exam scribing for team‐based documentation and between‐visit care coordination
Onsite specialty services	Clinical pharmacy services for diabetes, physical therapy, behavioral health
Protected provider time	Protected provider time for care coordination
Telehealth	Video and phone visits
Health coaching	Staff support of patient health goals through motivational interviewing techniques
Learning health care system structures	Including, but not limited to, continuous quality improvement, daily “huddles,” case conferences, and data monitoring

After extensive staff and provider training and planning, Stanford launched Primary Care 2.0 in June 2016 as a pilot in a new academic primary care clinic located in a community setting. The patient panel was initially about 1700 with a 3‐year goal of 10 000. The clinic serves a patient population that is diverse both ethnically and socioeconomically, with 72% non‐White patients and 14% publicly insured.

### 3.2 Pragmatic CFIR application

We divided the evaluation of Primary Care 2.0 into three stages: evaluation design, qualitative data collection and analysis, and assessment of spread. Here, we report on our utilization of CFIR in the first two stages, as stage 3 data collection and analysis of spread are currently in process. At each stage, CFIR served as a skeleton around which we structured our evaluation questions, methods, and reporting.

#### 3.2.1 Stage 1: Application of CFIR in evaluation design (November 2015‐May 2016)

In the first stage of our evaluation, we designed a plan appropriate for assessing key implementation outcomes in the context of a rapidly changing health system. We chose to frame our evaluation plan around CFIR as we expected it to be sensitive to most aspects of the complex implementation process. We used CFIR's five broad domains (intervention characteristics, outer setting, inner setting, characteristics of individuals, and process of implementation) to focus our evaluation measures by pairing them with the following implementation science outcomes: acceptability, adoption, appropriateness, feasibility, and adaptation. We used this combination of CFIR domains and implementation outcomes for each of the six modules of Primary Care 2.0 to drive our data collection, analysis, and reporting.

#### 3.2.2 Stage 2: Application of CFIR in qualitative data collection and coding (May 2016‐May 2018)

For the second stage of our application of CFIR, we collected and coded qualitative data. Table 2 summarizes the data collection methods and their relationship to the CFIR domains and implementation of the six modules of Primary Care 2.0. We used semi‐structured interviews and observations, as well as quantitative surveys, to collect data to assess implementation outcomes at the pilot site clinic. An additional file outlines the qualitative methods used in more detail. We structured interviews and observations around the five CFIR domains and included questions and observation guides drawn from online CFIR references.^2^

**Table 2:** Data collection methods for each Primary Care 2.0 module, by CFIR domain

	Team‐Based Care	Care Coordinator Role	Extended Care Team	Population Health Time	Telehealth	Health Coaching	Continuous QI
Intervention characteristics	• Observations • Interviews	• Observations • Interviews	• Observations • Interviews	• Interviews	• Observations • Interviews	• Observations • Interviews	• Observations • Interviews
Outer setting	• Interviews	• Interviews	• Interviews		• Interviews	N/A	• Document review
Inner setting	• Observations • Interviews • *Sustainability surveys*	• Observations • Interviews • *Sustainability surveys*	• Observations • Interviews • *Sustainability surveys*	• Interviews • *Sustainability surveys*	• Observations • Interviews • *Sustainability surveys*	N/A	• Document review • Interviews • *Sustainability surveys*
Characteristics of individuals	• Interviews • *Teamness and Wellness Surveys*	• Interviews • *Teamness and Wellness Surveys*	• Interviews • *Teamness and Wellness Surveys*	• Interviews	• Interviews	N/A	• Interviews
Process	• Interviews • Observations	• Interviews • Observations	• Interviews • Observations	• Interviews • Observations	• Interviews	N/A	• Interviews • Document review

*Note.* Rapid ethnography utilizes observations, informal interviews, and brief survey methodologies. Non‐italics refer to qualitative methods, while italics refer to quantitative methods.

Table 3 includes sample questions from our semi‐structured interview guides, by stakeholder group.

**Table 3:** Sample semi‐structured interview questions, by stakeholder group

Stakeholder Group	Question	CFIR Domain(s)
Stanford Health Care‐level implementation leadership (Level 3)	What are the drivers for introducing the model (internal/external)?	Outer setting, Inner setting
Peer primary care clinics (Level 2)	What do you know about Primary Care 2.0/Primary Care 2.0 spread?	Inner setting
Clinic‐level implementation leadership (Level 1)	There have been a lot of changes at your clinic, how has your team stayed resilient?	Inner setting
Pilot clinic providers and staff (Level 1)	How is the service and care offered through Primary Care 2.0 different from primary care offered elsewhere?	Intervention characteristics

Two qualitatively trained researchers (C.B.J. and N.S.) conducted five site visits over 2 years, utilizing rapid ethnography principles to embed into the pilot site clinic and observe implementation. Rapid ethnography draws upon multiple related data collection methods (eg, observations and semi‐structured interviews) in a short time frame while retaining a patient‐centered focus.^10^ We captured staff and provider experiences through brief, informal conversations and hour‐long semi‐structured interviews using a convenience sampling strategy. Interview guides included CFIR constructs across the original five domains to assess stakeholder understanding of Primary Care 2.0, its six modules, and any barriers and facilitators associated with implementation. Interview guides also included questions to capture stakeholder expectations of Primary Care 2.0 implementation across time. We captured data across all levels of the organization, including Stanford Health Care administrators, Stanford Primary Care leaders, and pilot site clinical leaders and staff.

Both of our field researchers completed field notes within 1 day of each site visit. Field researchers also conducted a 30‐ to 60‐minute debrief with each other at the end of each site visit day to cross‐reference implementation themes, insights, and lessons learned. We used CFIR as an informal checklist to organize findings during these qualitative data rapid synthesis debriefs. The debriefs also surfaced necessary changes and iterations needed in the data collection process.

We downloaded qualitative data, including 23 interview transcripts, into NVivo 11.4.2 for analysis. Transcripts included interviews from Stanford Health Care leadership (n = 2), pilot clinic leadership (n = 2), and on‐the‐ground staff and providers (n = 19). Two of the authors (C.B.J. and N.S.) collected, coded, analyzed, and synthesized all qualitative data. Using the 39 CFIR constructs as baseline codes, we developed a codebook to guide the coding process. We tailored definitions for each construct to fit the Primary Care 2.0 model. Coders C.B.J., a PhD qualitative expert, and N.S., an MPH‐trained qualitative researcher, developed and agreed upon adapted definitions. We also added additional codes for each module of the Primary Care 2.0 model and adapted operational definitions for each from the Primary Care 2.0 durable record.

Coders analyzed each data source for fit within each Primary Care 2.0 module and CFIR construct; a single data point could be, and often was, categorized to multiple constructs and modules. At periodic intervals, the researchers coded a transcript together to ensure inter‐coder agreement and reliability.

We coded transcripts using all 39 of CFIR's constructs and chose to explore each construct using deductive content analysis, an approach that utilizes a framework for analysis based on previous knowledge, 11 with each of CFIR's 39 constructs as primary nodes. Additionally, we included six modules of the Primary Care 2.0 model in the coding structure. We analyzed coded data using the “queries” function of NVivo, which generates counts of code incidence across all data. We used an analytic matrix, juxtaposing Primary Care 2.0 modules and relevant CFIR constructs, to identify overlap between the two frameworks. This process identified several CFIR constructs that did not yield any data, for example, trialability, external policies and incentives, and organizational incentives.

## 4 RESULTS

We made three key adaptations to CFIR at different stages throughout our evaluation: 1) promoted the “patient needs and resources” construct from the “outer setting” domain to its own sixth domain in the framework; 2) divided the “inner setting” domain into three layers to better reflect the complexity and hierarchy of implementation within a health care system; and 3) tailored CFIR construct definitions to fit the intervention's patient‐centered, team‐based care context and support consistency of data collection and coding.

### 4.1 A sixth domain: Patient needs and resources

The most significant change to CFIR structure arose in the process of coding of our qualitative data. Following our initial coding, we conducted a matrix analysis juxtaposing the five CFIR domains and each of the Primary Care 2.0 modules, as shown in Table 4. Reviewing the data at the intersection of these two categories sparked further analysis where high and low frequencies emerged, specifically within the outer setting domain which yielded the highest number of data points. A deeper dive revealed that a large portion of the “outer setting” data pertained to themes around patient satisfaction, needs, and resources. Our team promoted the CFIR construct “patient needs and resources” to sixth domain during analysis, due to the high frequency with which patients were referenced (a total of 46 times), as well as the categorical mismatch between CFIR's definition of the outer setting and the cross‐cutting nature of themes around patient needs and resources.

**Table 4:** Primary Care 2.0's six core modules mapped to CFIR's domains

CFIR Domains	Primary Care 2.0 Core Modules						Total
	Care Coordinator Role	Extended Care Team	Lean Leadership	Provider Time	Team‐Based Care	Telehealth	
Intervention characteristics	16	4	0	1	18	1	40
Outer setting^b^	0	1	0	0	2	0	3
*Patient needs and resources* a	17	11	0	3	10	5	46
Inner setting	2	3	0	0	9	0	14
Characteristics of individuals	7	4	0	0	5	0	16
Process	3	0	0	0	0	1	4

a Originally a construct in the domain of “outer setting.” We recommend promoting the construct to its own domain due to total counts.

b Outer setting does not include excerpts related to patient needs and resources.

### 4.2 Inner setting

**Figure 1:**
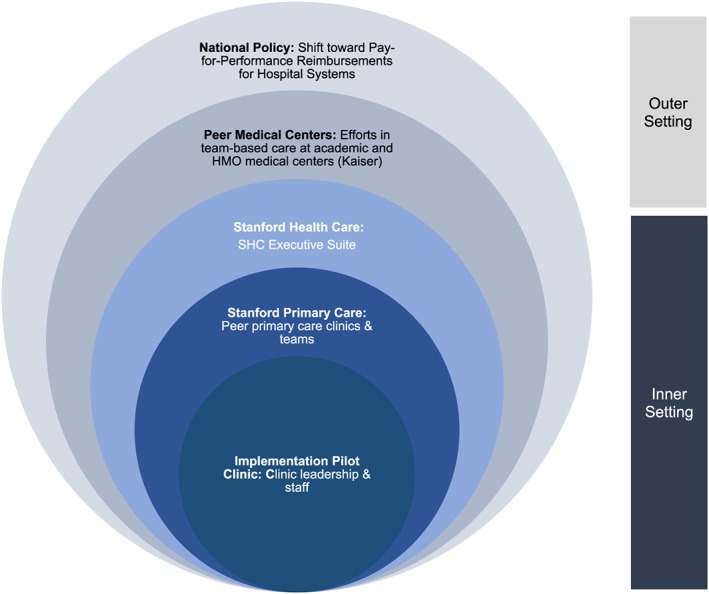
Key stakeholders and societal influences impacting Primary Care 2.0 implementation

The CFIR framework draws particular attention to the intervention setting, which it divides into inner and outer settings, in order to capture contextual forces on implementation. The “inner setting” refers to implementation factors as they exist within an “organization,” while the outer setting mostly considers influences external to the organization. Distinct from the original application of CFIR and as part of our data collection planning process, we further divided the inner setting into three distinct subparts, defined in Figure 1. These levels were adapted from organizational theory, which posits that effective efforts to impact quality consider four levels of change within a health system: the individual, the group or team, the organization, and the larger system and environment.^12^ We redefined CFIR's use of “organization” as the measure of inner setting and broke it down further in anticipation of the nuance between the experiences and perspectives of the different stakeholders and influencers involved in the transformation, including (a) the implementation pilot site and the on‐the‐ground clinic leadership and staff, (b) the network of peer academic primary care clinics and clinical teams within the organization, and (c) the overarching organization in which Primary Care 2.0 is housed. We defined the outer setting as peer academic medical centers, as well as the national health policy climate.

The structural, cultural, and readiness constructs defined within CFIR's inner setting are well‐suited for a simple, evidence‐based intervention within a small organization but do not attend to the complexity, hierarchy, and diverse stakeholders characteristic of a learning health care system. To effectively apply CFIR in the setting of our academic medical center, these stratified layers of inner setting, delineated by stakeholder groups and organization hierarchy, helped us identify the ways in which the different hierarchical components impacted the implementation process. Applying the inner setting constructs independently at these three levels brought our attention to the distinct implementation climates, variation in leadership engagement, patient needs, and cultures in each. Using these levels, we shaped our data collection tools and approach to purposively sample stakeholders across all three. Qualitative data that emerged showed some differences by group. For example, when asked “What does Primary Care 2.0 mean to you?”—a question designed to address the inner setting's “access to knowledge and information” subconstruct—medical assistants implementing the model at the pilot clinic (inner setting level 1) stated, “it's a better way of doing things … an attempt to resolve some of the issues with primary care … ,” whereas implementation leadership within Stanford Health Care administration (inner setting level 3) defined the transformation as “Stanford's version of the advanced medical home … in an effort to try to transform primary care into an activity that meets the quadruple aim.” Stakeholders at peer clinics (inner setting level 2) reported very minimal understanding of the transformation, with some team members stating, “I don't know … it's a little confusing to me and I'm not really sure how it's all gonna work.”

### 4.3 Tailored CFIR construct definitions

The first barrier our team encountered in applying CFIR was agreement for each construct's meaning and application in our specific intervention and clinical context as we developed our data collection materials. We found some constructs repetitive and others too broad in the context of an academic medical center. To address these issues, the primary researchers C.B.J. and N.S. reviewed each construct and codeveloped tailored definitions that were clear to both and a better fit for the context of a patient‐centered care transformation within a learning health system. Table 5 provides examples of these definitions.

**Table 5:** Sample CFIR constructs with tailored definitions

CFIR Construct	Definition	Tailored Definition
Goals and feedback (*Inner Setting*)	The degree to which goals are clearly communicated, acted upon, and fed back to staff, and alignment of that feedback with goals.	Clinic‐led communication and activities around implementation goals and progress in meeting them.
Reflecting and evaluating (*Process*)	Quantitative and qualitative feedback about the progress and quality of implementation accompanied with regular personal and team debriefing about progress and experience.	Stanford Health Care leadership‐led data collection, analysis, and reporting regarding implementation progress, with support from the evaluation team.
Champion (*Process*)	Individuals who dedicate themselves to supporting, marketing, and “driving through” an implementation, overcoming indifference or resistance that the intervention may provoke in an organization	Individuals outside of leadership roles (at the pilot clinic and administrative leadership levels) who are internally motivated to support implementation.
Patient needs and resources (*Outer Setting*)	The extent to which patient needs, as well as barriers and facilitators to meet those needs, are accurately known and prioritized by the organization	Any reference to patient needs, satisfaction, or feedback regarding the intervention, as reported by patients, caregivers, staff, or leadership.

For example, we addressed the overlap between the constructs “goals and feedback” and “reflecting and evaluating” by tailoring each to exist exclusively in different settings. We redefined “goals and feedback,” originally categorized to the “inner setting” domain, as communication and activities around implementation goals and progress that is led by the pilot clinic leadership. We characterized “reflecting and evaluating,” a construct of the “process” domain, as an exercise led by Stanford Health Care leadership with support from evaluation team efforts, as part of a partnered research approach. Although they originally exist within two different domains, the overlap between these constructs in the context of this transformation necessitated a shift of “goals and feedback” to the “process” domain since the new definition articulated a process to support implementation.

Much like the process for codebook development in preparation for qualitative data analysis, which emphasizes clear exclusion and inclusion criteria to support inter‐coder reliability,^13^ we aligned our working CFIR definitions at the start of the evaluation to ensure interobserver concordance.

Table 6 summarizes the adaptations to CFIR we made and the resulting value added. The most significant modification was the addition of a patient‐centered sixth domain to the framework, which elevated the need for patient priorities and voices to the forefront of implementation science work in health care settings. This grew out of the two preceding CFIR modifications that arose in our evaluation process: A more nuanced description of the inner setting supported identification of decisional and operational change at each level of implementation and tailored CFIR construct definitions that clarified and standardized qualitative data collection at each phase of the evaluation.

**Table 6:** CFIR adaptations and value added, by evaluation stage

Evaluation Stage	CFIR Innovations	Value Added
Stage 1: Evaluation design	Nuanced inner setting	Reflects the complexity and hierarchy of the health care system while facilitating more nuanced identification of drivers of decisional and operational change
Stage 2: Data collection	Tailored CFIR construct definitions	Allows for consistent data collection and analysis across researchers, and clarifies repetitive or vague CFIR construct definitions for future use
Stage 2: Data analysis	Additional domain: Patient needs and resources	Highlights the importance of patient needs and voices in patient‐centered care transformations and prioritizes focus on this domain during future evaluation design, data collection, and analysis

## 5 DISCUSSION

This study describes the pragmatic adaptations we made to CFIR to make it accessible and relevant for wider use in the context of patient‐centered care transformations. While CFIR has been applied in health care across a wide range of setting and objectives, it has rarely been combined with the complexity of implementing and evaluating a multifaceted restructuring of care. In their systematic review of CFIR application, Kirk et al highlight gaps in depth of use of the framework, variation in selection of CFIR constructs, and a lack of justification of constructs used, limiting comparison of findings across time and contexts.^14^ Our pragmatic and thorough use of all of CFIR's constructs, paired with tailored definitions, addresses these limitations and takes into consideration the underrepresented patient voice in the framework. Additionally, these adaptations facilitated a more streamlined application of CFIR well‐suited to the time frame and regular feedback channels of our rapid‐cycle evaluation.

The CFIR modifications we describe enabled us to have a deeper and richer understanding of issues that both facilitated and hindered implementation of each of the modules of Primary Care 2.0, with more robust consideration of patient and local health care system angles, than if we had used the framework in its original form. Primary Care 2.0 is a patient‐centered transformation effort and as such focuses heavily on patient needs, feedback, and satisfaction in each aspect of implementation. CFIR touches on the patient minimally in the construct “patient needs and resources” within the “outer setting” domain but insufficiently for this type of evaluation. Patient perspectives and experience do not feature largely in CFIR nor most studies that rely on it. One indicator of this is a finding by Kirk et al: Among 26 research studies that meaningfully used CFIR, only two of the 26 included patients among their unit of analysis.^14^

Our major adaptation of promoting the “patient needs and resources” construct to its own sixth domain in the framework recognizes the fact that health care interventions increasingly put patients and their families front and center, a focus that is given sparse attention in many implementation science frameworks. Focusing on patient needs as an additional dimension to the evaluation provided a richer picture of implementation and surfaced barriers and facilitators otherwise missed through use of the original CFIR with its five domains. The addition of a sixth domain may also ensure that future evaluations prioritize patient needs during evaluation design, data collection, and analysis.

Dividing the “inner setting” domain into three layers to better reflect the complexity and hierarchy of our health care system (ie, a primary care network within a broader academic health system that has strong tertiary and quaternary‐level enterprise) facilitated our identification of drivers of decisional and operational change. Whereas original use of CFIR would have us consider the inner setting for an organization overall, we harnessed lessons learned from organizational theory to pay attention to differences in implementation factors at various levels throughout. These factors were vital to understanding the evolution of Primary Care 2.0, especially when the health care system leadership changed midway through implementation. Health care administrative leadership changes are a common and ongoing state of health care, and it is wise to plan for them when implementing and evaluating change. Our adaptation of CFIR supports such planning, particularly for complex care transformations that are susceptible to impacts due to leadership changes. Thus, change drivers such as leadership changes became a fascinating part of the evaluation that would otherwise have been missed. Teasing out the layers of the inner setting was valuable to all stakeholders. On‐the‐ground pilot clinic voices were magnified, confidentially, up to those in leadership. Stanford Health Care leadership, in turn, learned of gaps in knowledge and resources at the pilot and peer clinic level that might not have otherwise been surfaced and were attuned to implementation barriers and facilitators through our team's regular rapid‐cycle feedback cycles structured around “lightning reports,” an actionable tool summarizing close‐to‐real time qualitative findings. In a learning health system context, drawing out the nuance of experiences at each level, from front‐line staff to C‐suite executives, supported our evaluation efforts and in turn strengthened our partnered‐research relationship by breaking down communication silos across the inner setting levels. Overall, the ability to describe and evaluate this dynamic system in a way that gave structure to the challenges and facilitators was effectively achieved by teasing out the different inner setting layers of the health care system hierarchy.

Lastly, our CFIR adaptation of tailoring and respecifying construct definitions was essential to fit them to our patient‐centered primary care clinical context and to allow consistent data collection and coding. Initially, using CFIR as a framework for data collection and analysis was cumbersome, time‐consuming, and difficult to standardize across researchers. This experience has been reported by other researchers applying the framework to complex evaluations and has been acknowledged by CFIR's creators.^1^, ^3^, ^4^, ^14^, ^15^ Primary barriers to CFIR use were the number of constructs, as well as vague and often repetitive construct definitions. Tailoring implementation frameworks—like CFIR—may improve intervention outcomes^16^; however, few implementation studies appropriately match their implementation and evaluation strategies to contextual factors.^17^ Effective CFIR application required innovative adjustments specific to a learning health system environment, such as tailored construct definitions made more broadly applicable to the transformation initiative, to incorporate the framework effectively in the context of partial implementation and ongoing adaptation of the model.

There are two limitations to this research. First, we used a subset of qualitative data collected during the evaluation period in this analysis. We used a subset in order to facilitate rapid feedback to Stanford Health Care leadership, but we also were attentive to the need to reach theme saturation, which is a standard approach in rapid qualitative analysis that we achieved in this study.^10^ Second, we were not able to include patient interviews and observations we conducted in this analysis due to insufficient sample size. Both limitations are artifacts of project resourcing.

While others have also used CFIR in evaluating care transformations and found the framework valuable in their efforts,^4^, ^5^, ^6^ we are the first to add depth to the “inner setting” domain and recognize the need to elevate patient‐facing elements of redesign by making “patient needs and resources” its own domain rather than a construct within a domain. Supported by community‐engaged research and other user‐centered approaches, we believe the prioritization of and attention to the patient in this adaptation is essential and generalizable to implementation of any human‐centered intervention in a health care setting. A patient‐centered CFIR is also well‐positioned to complement the growing body of clinical effectiveness and implementation hybrid research studies.^18^ As a whole, these adaptations to CFIR facilitated robust data collection and analysis across multiple qualitative researchers in a patient‐centered, outpatient primary care health setting. We also hypothesize that they are applicable to other patient‐centered, outpatient contexts. Our adaptations may also be transferrable to a wider array of audiences and fields, including user‐centered design, academia, and community organizations.

## 6 CONCLUSION

We believe this example of how to tailor CFIR will make CFIR more accessible and relevant for wider use in the context of patient‐centered health care interventions, especially within the context of multi‐part health care systems. Incorporating the adapted CFIR into our evaluation allowed us to assess a complex, dynamic primary care initiative while capturing context and culture‐specific factors that influenced implementation across each level of the organization. We found CFIR to be a powerful longitudinal evaluation tool that facilitated capture of thoughtful nuances and key voices throughout the implementation process. Adaptation of the framework, however, was key for successful pragmatic application.

Placing additional emphasis on patient needs and resources; adapting the “inner setting” domain to reflect the complexity of health care organizations; and developing tailored, agreed‐upon definitions for CFIR constructs at the start of the evaluation supported our team in capturing real‐time changes within the implementation process and filled significant gaps in the framework. We believe our adaptations will be helpful to others and encourage wider application of CFIR and these strategies to move forward the science of evaluating patient‐centered health care redesign.

## LIST OF ABBREVIATIONS

CFIR: Consolidated Framework for Implementation Research

## CONFLICTS OF INTEREST

Author Mahoney is currently a practicing provider in Stanford Health Care's system. We have no other conflicts of interest to report.
